# Dimensions of Masculine Norms, Depression, and Mental Health Service Utilization: Results From a Prospective Cohort Study Among Emerging Adult Men in the United States

**DOI:** 10.1177/1557988320906980

**Published:** 2020-02-21

**Authors:** Katelyn M. Sileo, Trace S. Kershaw

**Affiliations:** 1 The University of Texas at San Antonio, TX, USA; 2 The Center for Interdisciplinary Research on AIDS, Yale University, New Haven, CT, USA; 3 Department of Social and Behavioral Sciences, Yale School of Public Health, New Haven, CT, USA

**Keywords:** Depression, mental health, help seeking, masculinity, gender norms, men

## Abstract

The purpose of this study was to examine the role of multidimensional masculine norms (“status,” “toughness,” “anti-femininity”) on depression and mental health service utilization among emerging adult men in the Northeast United States. This study examines substance use and hostility as secondary outcomes and depression status as an effect moderator on the relationship between masculine norms and mental health service utilization. This study used data from a prospective cohort study that followed 18- to 25-year-old heterosexual men over 6 months. At baseline and 6 months, approximately 29% and 25% of the sample met the criteria for depression. The results of multivariate linear and logistic regression models found that greater endorsement of masculine status was associated with less depressive symptoms at baseline and 6 months, masculine toughness was associated with more substance use at baseline, and masculine anti-femininity was associated with greater hostility at baseline and 6 months. The multivariate Poisson model found that greater endorsement of status was associated with greater mental health service utilization in the prior year, especially for men not meeting the criteria for depression. In contrast, greater endorsement of anti-femininity and toughness norms was associated with less mental health service utilization; for men endorsing toughness norms, this effect was greater for those who were depressed. This study sheds light on the harmful and protective effects of masculine norms on depression, related mental health outcomes, and mental health service utilization, with implications for gender-tailored approaches to engage and retain young men in mental health services.

The World Health Organization ranks depression as the leading cause of disability worldwide, affecting approximately 300 million people globally (World Health Organization, 2018). In the United States, an estimated 7.1% of adults, or 17.3 million people, had at least one major depressive episode in 2017 (National Institute of Mental Health, 2019). Though women are consistently reported to be at greater risk for depression than men (Albert, 2015; Kessler & Bromet, 2013; National Institute of Mental Health, 2019), the burden of depression among men is likely underestimated, as men are less likely to seek treatment. In a national survey, nearly 9% of men had daily feelings of anxiety or depression, but less than one half of men (41.0%) took medication for these feelings or had recently talked to a mental health professional (Blumberg et al., 2015). Men of younger age, men of color, and those with lower socioeconomic status are even less likely to seek mental health services and receive treatment when needed (Blumberg et al., 2015; Chandra et al., 2009; Cummings, 2014; National Institute of Mental Health, 2019; Parent et al., 2018). Suicide is 3.6 times greater among men compared to women and is the second leading cause of death for males aged 10–34 years (Hedegaard et al., 2018; National Center for Injury Prevention and Control, 2017). Men report higher rates of anger, aggression, substance abuse, and risk-taking compared to women, which may be male-specific symptoms of depression that go unrecognized as such (Call & Shafer, 2018; Martin et al., 2013; Rochlen et al., 2010).

Masculine norms, or the culturally grounded expectations for men’s roles, behaviors, and relationships, are a driver of men’s mental health status and health-seeking behavior (Courtenay, 2000a, 2000b). From a social constructionist perspective, Courtenay (2000a, 2000b) argues that health beliefs and behaviors are a display of masculinities and femininities and reinforce the broader social structure of gender and power. Adherence to North American ideals of masculinity requires the rejection of femininity and weakness, which shapes men’s health attitudes and behaviors (Courtenay, 2000a, 2000b). This idea extends to how men respond to depressive symptoms and engage with mental health services (Addis & Mahalik, 2003; Mahalik & Rochlen, 2006; Smith et al., 2018). The socialization of men to be strong, resilient, independent, and emotionally inexpressive and to avoid weakness and femininity contributes to the masking of depressive symptoms among men as well as men’s delay in or avoidance of treatment seeking (Johnson et al., 2012; Keohane & Richardson, 2018; Oliffe et al., 2011; Seidler et al., 2016). In addition to affecting men’s engagement with psychological health services, adherence to traditional masculine norms is thought to directly impact men’s mental health status. The dysfunction strain paradigm is a framework often used to understand this relationship, positing that the pressure men feel to fulfill often unattainable societal gender norm expectations causes “masculine strain” that can lead to adverse psychological outcomes (Pleck, 1981, 1995). In support of this theory, research has demonstrated associations between adherence to traditional masculine norms and depression, anxiety, hostile behaviors, and other adverse mental health outcomes (Gerdes & Levant, 2018; Levant et al., 2013; O’Neil, 2008; Seidler et al., 2016; Vogel et al., 2011).

Quantitative research linking masculine norms with poor mental health and men’s health behaviors more broadly, however, has been based primarily on cross-sectional studies using a composite measure of adherence to traditional masculine norms (Gerdes & Levant, 2018; Wong et al., 2017). Our understanding of masculinity is not one of a static, singular definition, but is multidimensional, dynamic, and adaptable across different social situations and cultural contexts (Connell, 1995; Connell & Messerschmidt, 2005; Courtenay, 2000a; Mankowski & Maton, 2010). Gerdes et al.’s (2018) content analysis of 17 published studies demonstrated how the study of a single measure of masculinity obscures more complex relationships between conformity to specific masculine norms and men’s health and well-being, finding both positive and negative effects of specific masculine norms on health outcomes and psychological treatment seeking when taking a nuanced approach to analysis.

Wong et al.’s (2017) meta-analysis similarly demonstrated an overall negative effect on mental health outcomes with a composite measure but distinct patterns when disaggregated into different dimensions or distinct domains of socially constructed masculinity (Levant et al., 2015). Conformity to dimensions of “self-reliance,” “power over women,” and “playboy” (i.e., desire to have multiple sexual partners) were consistently associated with unfavorable mental health–related outcomes, whereas conformity to the masculine norm of “primacy of work” was not significantly related to any mental health–related outcome. Specific to depression, a recent prospective study among college men reported greater risk for depression among men who endorsed the masculine norms of “self-reliance,” “playboy”, and “violence” and less depressive symptomatology among those endorsing “winning” and “power over women” (Iwamoto et al., 2018). “Winning” and the related construct of “status” (i.e., striving for success and power) have been positively associated with other positive health outcomes and treatment-seeking behavior (e.g., less substance use, healthy diet, use of general health services; Gordon et al., 2013; Salgado et al., 2019). Hypothesized explanations include more preventative self-care, better coping strategies, and more resilience and self-esteem among men endorsing these masculine traits (Gerdes & Levant, 2018; Gordon et al., 2013; Salgado et al., 2019), but more research is need to understand the protective versus harmful effects of masculine norms specific to mental health outcomes.

This literature highlights the need for more research that examines the population- and context-specific multidimensional effect of masculine norms on depression, related mental health outcomes, and mental health service engagement. Only one study to date has used longitudinal data to understand masculine norms and depression (Iwamoto et al., 2018), highlighting the need for more prospective studies with this aim. The specific aims of this study were to explore the effect of dimensions of masculine norms (“status,” “toughness,” “anti-femininity”) on (a) depression, substance use, and hostility and (b) mental health service utilization among an ethnically and racially diverse sample of emerging adult men from low-income neighborhoods. This article focuses on masculine status (i.e., striving toward competition, success, and power), toughness (being physically and mentally tough), and anti-femininity (the rejection of femininity) (Mankowski & Maton, 2010; O’Neil et al., 1986); though not exhaustive, these are among the most commonly studied dimensions of masculinity. Substance use and hostility are included as secondary outcomes to capture a broader definition of men’s depression based on evidence that standardized measures of depressive symptoms do not assess externalizing symptoms of depression common among men, such as alcohol and drug use, aggression, anger, irritability, emotional suppression, and somatic symptoms (Call & Shafer, 2018; Martin et al., 2013; Rochlen et al., 2010). Though not inclusive of the full range of male-specific symptomology, substance use (including alcohol and other drug use) and hostility (which captures anger, aggression, and irritability) are included to assess externalizing symptomology.

Based on prior studies, it was hypothesized that status would be associated with better mental health outcomes and service utilization, while toughness and anti-femininity would be associated with worse outcomes and utilization. This study’s inclusion of a diverse sample of emerging adult men extends the current literature on masculinity and mental health, in which minority populations from lower income communities are underrepresented. This population is pertinent to the stated research questions, given low mental health treatment seeking (Blumberg et al., 2015; Chandra et al., 2009; Cummings, 2014; National Institute of Mental Health, 2019; Parent et al., 2018) and high stigma associated with mental health services among individuals from low-income settings (Vogel et al., 2011). In addition to the above-stated research questions, depression status is examined as an effect moderator on the relationship between masculine norms and mental health service utilization. This research question is intended to shed light on the role of dimensions of masculinity in primary versus secondary prevention behavior as it relates to mental health seeking behavior, which has not been previously examined. That is, how do masculine norms affect health-seeking behavior among young men who do not meet the criteria for depression (primary prevention) compared to those who do meet the criteria for depression (secondary prevention)?

## Methods

This analysis was conducted with data from a longitudinal cohort study that followed 119 emerging adult men (ages 18–25 years) over a 6-month period to assess social networks, cell phones, and health behavior (Gibson et al., 2015). Men were recruited in networks using snowball sampling in a small urban area in the Northeast, United States. Neighborhoods with high levels of negative structural and social determinants of health (e.g., crime, sexually transmitted infections, and poverty) were first identified based on epidemiological assessments of U.S. Census and State Health Department Data. The study team then conducted ethnographic mapping of neighborhoods to identify areas where emerging adult males frequented for targeted recruitment by outreach workers. The outreach workers visited these locations to share information about the study and their contact information. Interested potential participants called or directly approached the outreach worker, who informed them of the study and obtained written informed consent. Using snowball sampling, men referred friends to the outreach worker and received $10 for each participant they referred.

Participants were screened for eligibility over the phone or in person. Eligibility criteria included (a) male gender; (b) age 18–25 years; (c) English speaking; (d) heterosexual; (e) ownership of a cell phone with texting capabilities; and (f) ability to maintain cell phone service. The original study was specific to high-risk heterosexual men; focusing on a homogeneous population was appropriate, given the small nature of the study and differential risk profiles and predictors based on sexual orientation. Each participant completed an Audio Computer-Assisted Self Interview (ACASI) that collected self-reported information on demographics, health behaviors, and attitudes at enrollment and 3-month and 6-month follow-ups, with $75 compensation for the completion of each computerized interview. All procedures were approved by the Yale University Human Subjects Committee. Of the 119 men enrolled in the study, 2 men did not complete the measures on masculine role norms and were therefore excluded from this analysis, making the total sample size for this article 117. In addition to the baseline data, this article includes data collected at 6-month follow-up, which had better retention (87%) than the 3-month assessment (72%).

### Measures

Of the sociodemographics measured, the following were included as potential covariates in analysis: age (continuous), race/ethnicity (Black or African American, Hispanic/Latino, White, and multiracial), education (categorized for analysis based on distribution as 11th grade or less, high school diploma or general equivalency diploma [GED], and at least some college), and medical insurance (yes/no). Perceived stress was included as a covariate, as it correlated with the study’s mental health outcomes, measured using Cohen and Williamson’s (1988) 10-item Perceived Stress Scale (PSS). Participants indicated how often in the past month they had experienced stressful feelings and thoughts, for example, “felt upset by something that happened unexpectedly” and “felt nervous or stressed.” Response options were a 5-point scale ranging from 0 = *Never* to 4 = *Very often*. Cronbach’s α in the current sample = 0.76 at baseline and 0.86 at 6 months. Anxiety control was also included as a covariate, measured using Brown et al.’s (2004) revised Anxiety Control Questionnaire (ACQ). The 30-item instrument is designed to assess perceived control over emotional reactions, stress, and external threats, for example, “I am able to control my level of anxiety” and “I always know exactly how I will react to difficult situations.” Response options range from 0 = *Strongly disagree* to 5 = *Strongly agree*. Possible scores range from 0 to 150, with greater scores indicative of greater control of anxiety. Cronbach’s α in the current sample = 0.76 at baseline and 0.86 at 6 months.

Masculine role norms were measured at baseline with 25 items from the Masculine Role Norm Scale (MRNS), developed by Thompson and Pleck (1986).The MRNS is separated into three subscales to assess the degree to which men agree with statements that men should (a) acquire skills that warrant respect and admiration (11-item “status” norm subscale), (b) be mentally and physically tough (8-item “toughness” norm subscale), and (c) avoid anything feminine (6 items from the “anti-femininity” norm subscale, 1 item omitted due to wording that was not fitting for the population as determined by study team members with expertise on masculinity and the population of interest). Participants were asked to respond using a 7-point Likert scale ranging from 1 = *Strongly disagree* to 7 = *Strongly agree*. Possible scores for each subscale are as follows: status: 11–77, toughness: 8–57, and anti-femininity: 6–42. An earlier study reported good reliability across the MRNS subscales among minority males aged 15–25 years, a sample similar to the present study (Gordon et al., 2013). Cronbach’s α for the current study sample are as follows: status = 0.86, toughness = 0.71, and anti-femininity = 0.57.

The three mental health variables were measured at baseline and 6-month follow-up, including depression, substance use, and hostility. Depression, the primary outcome, was measured using the 20-item Center of Epidemiological Studies-Depression Scale (CES-D; Radloff, 1977). For each symptom of depression, participants indicated how often they felt or behaved in the specified way, ranging from 0 = *Less than 1 day a week* to 3 = *Most of the time (5-7 days a week)*. The total score was summed, with possible scores ranging from 0 to 60 and was used in analysis as a continuous variable. To test depression as an effect modifier in the model predicting mental health service utilization, depression was dichotomized using a widely accepted cutoff point of 16 or more to classify patients with depressive symptoms (Radloff, 1977; Weissman et al., 1977). Patients can be categorized into one of the following four groups based on their scores: not depressed (0–9 points), mildly depressed (10–15 points), moderately depressed (16–24 points), or severely depressed (more than 25 points) (Radloff, 1977; Weissman et al., 1977). Therefore, those classified as depressed in the present study met the criteria for moderate-to-severe depression. Cronbach’s α for the current study sample = 0.83 at baseline and 0.84 at 6 months.

Participants were asked about their lifetime substance use, as well as use in the prior 30 days using the NIDA-Modified ASSIST (National Institute on Drug Abuse) for the following substances: alcohol, marijuana, cocaine, glue/paint/spray cans, steroids, prescription drugs, heroin, ecstasy, methamphetamines, LSD, and mushrooms. For analysis, substance use was dichotomized into any substance use in the prior 30 days (yes or no). Substances reported in the present sample included alcohol, marijuana, cocaine, ecstasy, and prescription drugs. All participants who reported drinking alcohol in the prior 30 days also reported using marijuana and the prevalence of all other drug use was low; therefore, it was not possible to differentiate between different types of substance use in analysis (details on the prevalence for each substance are reported under results).

Hostility was measured using a 5-item, adapted version of the Brief Symptom Inventory (BSI) that asked participants how much, in the past 7 days, they were bothered by hostile or violent feelings, thoughts, or urges. The scale is comprised of the following items: “feeling easily annoyed or irritated,” “temper outbursts that you could not control,” “having urges to beat, injure, or harm someone,” “having urges to break or smash things,” and “getting into frequent arguments.” Participants responded on a 5-point scale ranging from 1 = *Not at all* to 5 = *Extremely*. Possible scores range from 5 to 20. Cronbach’s α in the current sample = 0.85 at baseline and 0.87 at 6 months.

To measure mental health service utilization, participants were asked the number of times they saw a mental health professional in the prior year at baseline, including a therapist or psychiatrist and a social worker. The total number was tallied into a count variable.

### Data Analysis Plan

All analyses were carried out using SPSS version 24. Analyses included baseline and 6-month follow-up measurements. Descriptive statistics and bivariate analyses were conducted between sociodemographic and mental health variables (age, race/ethnicity, education, medical insurance, perceived stress, anxiety control, depression, substance use, and hostility) with masculine norm variables as outcomes (status, toughness, anti-femininity). For 17 men (14.5% of the sample) who did not complete the 6-month assessments, missing values were imputed using their 3-month scores when possible, and if not available, their baseline scores for the following variables: depression, substance use, hostility, perceived stress, and anxiety control.

To assess the effect of masculine role norms on depression (primary outcome) and substance use and hostility (secondary outcomes), separate multivariate generalized linear models for depression and hostility and a logistic model for substance use were used for both baseline and 6-month follow-up time points. Each model was adjusted for age, education, health insurance, perceived stress, anxiety control, and the other mental health variables, as these were identified as covariates in bivariate analyses with at least one masculine norm variable. Using Poisson loglinear generalized linear modeling, the association between masculine status, toughness, and anti-femininity and the number of times men visited a mental health professional in the prior year was examined. This relationship was not examined prospectively as too few men reported mental health service utilization at the prospective points, in part due to missing data on this outcome. The model controlled for age, education, health insurance, as well as this study’s mental health outcomes (baseline assessment). Perceived stress, anxiety control, and race/ethnicity were dropped from the mental health service utilization model due to collinearity and model stability; they were not statistically significant in the final model. For masculine norms that were associated with health-seeking behavior (*p* < .05), interactions were tested between masculine norms and depression, dichotomized as meeting the criteria for depression (yes/no). This allowed for the examination of whether masculine norms affect health-seeking behavior differently for men with symptoms of depression versus those without. Betas (β) and standard errors (*SE*s) as well as adjusted odds ratios (AORs) and 95% confidence intervals (CIs) are presented and used to interpret effect size.

## Results

Sociodemographic characteristics of the sample are displayed in Table 1. Table 2 displays the bivariate correlations between these sociodemographic characteristics and mental health variables with masculine norms. Men on average were approximately 21 years old (*SD* = 1.98), ranging from 18 to 25 years of age. Men’s self-reported race/ethnicity were as follows: Black or African American (*n* = 74, 63.20%), Hispanic/Latino (*n* = 16, 13.70%), White (*n* = 5, 4.30%), and multiracial (*n* = 22, 18.8%). Of the 22 men identifying as multiracial, 8 men identified as Black and American Indian or Alaska Native, 7 as Black and Hispanic, 4 as Black and White, 2 as Hispanic and White, 1 as Black and Native Hawaiian or Pacific Islander. Nearly half of the sample reported having a yearly household income of less than $10,000 (*n* = 46, 45.50%); while 18.80% (*n* = 19) reported $10,000–19,999, 13.90% (*n* = 14) reported $20,000–$34,999, 9.90% (*n* = 10) reported $35,000–$29,999, and 11.90% (*n* = 12) reported $50,000 or greater. Most men (*n* = 92, 78.6%) reported having medical insurance.

**Table 1.** Participant Characteristics and Descriptive Statistics, *N* = 117.

	*n* (%)/mean (*SD*)	Range
Age	20.65 (1.98)	18–25
Race		
Black or African American	74 (63.20%)	
Hispanic or Latino	16 (13.70%)	
White	5 (4.30%)	
Multiracial	22 (18.8%)	
Household yearly income		
$0–$9,999	46 (45.50%)	
$10,000–$19,999	19 (18.80%)	
$20,000–$34,999	14 (13.90%)	
$35,000–49,000	10 (9.90%)	
$50,000 or greater	12 (11.90%)	
Highest grade completed		
11th grade	21 (17.9%)	
High school or GED	45 (38.5%)	
At least some college	51 (43.6%)	
% with medical insurance	92 (78.6%)	
**Masculine norms/roles (baseline)**		
Status	59.74 (12.17)	11–77
Toughness	38.27 (8.67)	15–57
Anti-femininity	22.59 (6.48)	7–39
**Mental health**		
Perceived stress (baseline)	15.30 (6.13)	1–37
Perceived stress (6 months)	13.84 (6.79)	0–39
Anxiety control (baseline)	92.95 (20.82)	2–142
Anxiety control (6 months)	93.30 (21.86)	0–153
Depression (baseline)	12.40 (8.47)	0–45
Depression (6 months)	11.87 (8.62)	0–47
% meeting the criteria for depression (baseline)	34 (29.10%)	
% meeting the criteria for depression (6 months)	29 (24.80%)	
Substance use in the prior 30 days (baseline)	70 (59.80%)	
Substance use in the prior 30 days (6 months)	68 (58.10%)	
Hostility (baseline)	3.82 (4.34)	0–18
Hostility (6 months)	3.89 (4.49)	0–20
**Mental health service utilization (prior year)**		
% saw mental health professional	16 (13.70%)	
# times saw mental health professional	2.71 (13.32)	0–100

*Note*: Criterion for depression is defined as a score of 16 or greater on the Center for Epidemiological Studies-Depression (CESD); substance use in the prior 30 days is defined as any alcohol, marijuana, or other drugs; other drugs reported included cocaine, ecstasy, and prescription pills; data for household yearly income missing for *n* = 16; GED = general equivalency diploma.

**Table 2.** Bivariate Associations Between Sociodemographic and Mental Health Variables and Masculine Norms, *N* = 117.

	Status			Toughness			Anti-femininity		
	β	*SE*	*p*	β	*SE*	*p*	β	*SE*	*p*
Age	–0.44	0.57	.44	0.30	0.40	.66	–0.71	5.19	**.01**
Race/ethnicity									
Multiracial	–2.44	5.85	.68	1.18	4.27	.78	–6.48	3.11	**.04**
Black or African American	0.61	5.46	.91	0.20	3.98	.96	–5.64	2.90	.05
Hispanic	–7.18	6.05	.24	–0.56	4.42	.90	–3.11	3.22	.33
White (reference)									
Education									
Some college or greater	4.55	3.11	.14	–0.43	2.24	.85	–1.52	1.62	.35
High school or GED	3.96	3.17	.21	–1.04	2.28	.65	–4.17	1.65	**.01**
11th grade (reference)									
Medical insurance									
Yes	0.13	2.73	.96	2.48	1.93	.20	2.94	1.43	**.04**
No (reference)									
Perceived stress (baseline)	–0.19	0.18	.29	–0.20	0.13	.12	0.01	0.10	.97
Perceived stress (6 months)	–0.21	0.16	.21	–0.34	0.11	**.003**	–0.04	0.09	.63
Anxiety control (baseline)	0.14	0.05	**.006**	0.09	0.04	**.02**	**–**0.11	**0.03**	**<.001**
Anxiety control (6 months)	0.15	0.05	**.004**	0.11	0.04	**.002**	–0.05	0.03	.09
Depression (baseline)	–0.31	0.13	**.02**	–0.15	0.09	.12	0.01	0.07	.85
Depression (6 months)	–0.36	0.12	**.004**	–0.33	0.09	**<.001**	–0.13	0.07	.06
Substance use (baseline)	3.16	2.27	.16	3.55	1.59	**.03**	–0.76	1.21	.53
Substance use (6 months)	1.42	2.27	.53	1.21	1.61	.45	–0.71	1.21	.56
Hostility (baseline)	–0.10	0.26	.71	–0.08	0.18	.66	0.31	0.13	**.02**
Hostility (6 months)	–0.25	0.25	.31	–0.45	0.17	**.01**	–0.04	0.12	.75
Mental health service utilization	0.52	0.08	.53	–0.05	0.06	.37	–0.01	0.05	.97

*Note*. β = beta, GED = general equivalency diploma; *SE* = standard error.

Boldface text indicates *p* values < .05.

Of the three masculine role norm subscales, status norms had the highest endorsement (mean = 59.73, *SD* = 12.17), followed by toughness (mean = 38.27, *SD* = 8.67) and anti-femininity (mean = 22.59, *SD* = 6.48). At baseline and 6-month follow-up, approximately 29% and 25% of the sample met the criteria for depression using the CES-D measure. Nearly 60% of participants reported substance use in the prior 30 days at both baseline (*n* = 70) and 6-month follow-up (*n* = 68). Of those reporting substance use, 100% reported both alcohol and marijuana use at baseline and 6 months. The prevalence of other drug use in the prior 30 days was low at both time points (baseline: cocaine [*n* = 1], ecstasy [*n* = 3], prescription drugs [*n* = 3]; 6 months: ecstasy [*n* = 1], prescription drugs [*n* = 2]). Hostility scores were moderate (baseline: mean = 3.82, *SD* = 4.34; 6 months: mean = 3.89, *SD* = 4.49). Nearly 14% of the sample reported having seen a mental health professional in the prior year at baseline. The average number of visits in which the sample saw a mental health professional over the prior year was 2.71 (*SD* = 13.32).

### Results of Multivariate Linear and Logistic Regression Analyses Testing the Associations Between Dimensions of Masculine Role Norms and Mental Health Outcomes at Baseline and 6-month Follow-up

In the multivariate models examining associations with depression scores (see Table 3), greater endorsement of masculine status was negatively associated with depression at baseline (β = −0.11, *SE* = 0.05, *p* = .02) and at 6 months (β = −0.11, *SE* = 0.05, *p* = .02). Masculine toughness and anti-femininity were not associated with depression at either time point at a statistically significant level. Of the included covariates, having greater education (high school or GED: β = 3.81, *SE* = 1.52, *p* = .01; at least some college: β = 3.86, *SE* = 1.54, *p* = .01), more perceived stress (β = 0.75, *SE* = 0.10, *p* < .001), less anxiety control (β = −0.08, *SE* = 0.03, *p* = .02), and greater hostility (β = 0.41, *SE* = 0.14, *p* = .003) were associated with greater depressive symptoms at baseline. At 6 months, having no medical insurance (β = −3.27, *SE* = 1.28, *p* = .01), more perceived stress (β = 0.66, *SE* = 0.10, *p* < .001), and greater hostility (β = 0.38, *SE* = 0.14, *p* = .004) were predictive of greater depressive symptoms.

**Table 3.** Results of Multivariate Linear Regression Analyses Testing the Association Between Dimensions of Masculine Norms (Baseline) and Depression (Baseline, 6 Months), *N* = 117.

	Depression					
	Baseline			6 months		
	β	*SE*	*p*	β	*SE*	*p*
Age	–0.11	0.30	.70	0.02	0.27	.94
Race/ethnicity						
Multiracial	1.11	2.86	.70	1.74	2.73	.52
Black/African American	–0.72	2.62	.78	1.92	2.49	.44
Hispanic	–1.08	2.93	.71	2.52	2.80	.37
White (reference)						
Highest education						
Some college or greater	3.86	1.54	**.01**	2.24	1.49	.13
High school or GED	3.81	1.52	**.01**	2.51	1.48	.09
11th grade (reference)						
Medical insurance						
Yes	–1.38	1.34	.30	–3.27	1.28	**.01**
No (reference)						
Perceived stress	0.75	0.10	**<.001**	0.66	0.10	**<.001**
Anxiety control	–0.08	0.03	**.02**	–0.06	0.03	.06
Substance use	–0.19	1.22	.88	–1.17	1.02	.25
Hostility	0.41	0.14	**.003**	0.38	0.14	**.004**
Status	–0.11	0.05	**.02**	–0.11	0.05	**.02**
Toughness	0.07	0.07	.30	0.01	0.07	.94
Anti-femininity	–0.09	0.10	.37	–0.16	0.08	.06

*Note*. β = beta, *SE* = standard error.

Boldface text indicates *p* values < .05.

In the logistic regression model testing the associations between masculine norms and substance use (see Table 4), endorsing masculine toughness was associated with greater odds of reporting substance use in the prior 30 days at baseline (AOR = 1.08, 95% CI = 1.01–1.16, *p* = .03). Substance use did not relate at a statistically significant level to endorsement of any other masculine norm at either time point. Covariates with substance use identified at baseline included older age (AOR = 1.60, 95% CI = 1.20–2.12, *p* = .001), greater perceived anxiety control (AOR = 1.04, 95% CI = 1.01–1.07, *p* = .02), and greater hostility (AOR = 1.21, AOR = 1.05–1.40, *p* = .009).

**Table 4.** Results of Multivariate Logistic Regression Analyses Testing the Association Between Dimensions of Masculine Norms (Baseline) and Substance Use (Baseline, 6 Months), *N* = 117.

	Substance use (prior 30 days)					
	Baseline			6 months		
	AOR (95% CI)	Wald χ^2^	*p*	AOR (95% CI)	Wald χ^2^	*p*
Age	1.60 (1.20–2.12)	10.43	**.001**	1.16 (0.93–1.45)	1.76	.19
Race/ethnicity						
Multiracial	2.24 (0.15–34.39)	0.34	.56	2.31 (0.26–20.68)	0.56	.46
Black/African American	1.36 (0.11–16.91)	0.06	.81	1.90 (0.26–14.14)	0.39	.53
Hispanic	26.40 (1.04–672.51)	3.93	.05	10.08 (0.90–112.54)	3.52	.06
White (reference)						
Highest education						
Some college or greater	1.31 (0.33–5.17)	0.15	.70	1.17 (0.50–5.83)	0.74	.39
High school or GED	1.68 (0.42–6.70)	0.54	.46	1.18 (0.52–6.26)	0.86	.35
11th grade (reference)						
Medical insurance						
Yes	0.82 (0.24–2.78)	0.11	.75	0.49 (0.16–1.46)	1.65	.20
No (reference)						
Perceived stress	1.06 (0.94–1.18)	0.91	.34	1.09 (0.99–1.20)	2.95	.09
Anxiety control	1.04 (1.01–1.07)	5.46	**.02**	1.00 (0.98–1.03)	0.05	.83
Depression	1.01 (0.92–1.11)	0.04	.84	0.96 (0.89–1.03)	1.24	.27
Hostility	1.21 (1.05–1.40)	6.77	**.009**	0.98 (0.87–1.10)	0.13	.72
Status	1.02 (0.97–1.07)	0.74	.39	1.01 (0.97–1.05)	0.16	.69
Toughness	1.08 (1.01–1.16)	4.87	**.03**	1.03 (0.97–1.08)	0.78	.38
Anti-femininity	1.00 (0.91–1.09)	0.007	.93	0.99 (0.92–1.06)	0.13	.72

*Note.* AOR= adjusted odds ratio, 95% CI = 95% confidence interval; GED = general equivalency diploma.

Boldface text indicates *p* values <.05.

In the multivariate models with hostility as the outcome (see Table 5), endorsement of anti-femininity norms was associated with greater hostility at baseline (β = 0.13, *SE* = 0.06, *p* = .04) but was not associated with any masculine norm variables at 6 months. Greater depression was associated with higher hostility at both baseline (β = 0.17, *SE* = 0.06, *p* = .003) and 6 months (β = 0.17, *SE* = 0.06, *p* = .004). Men who reported using any substances in the prior 30 days also reported higher hostility at baseline (1.90, *SE* = 0.77, *p* = .01).

**Table 5.** Results of Multivariate Linear Regression Analyses Testing the Association Between Dimensions of Masculine Norms (Baseline) and Hostility (Baseline, 6 Months), *N* = 117.

	Hostility					
	Baseline			6 months		
	β	*SE*	*p* value	β	*SE*	*p* value
Age	0.06	0.19	.77	0.12	0.18	.49
Race/ethnicity						
Multiracial	–1.79	1.84	.33	–0.10	1.81	.96
Black/African American	–1.43	1.69	.40	–1.35	1.65	.41
Hispanic	–1.55	1.89	.41	–0.44	1.86	.81
White (reference)						
Highest education						
Some college or >	–0.87	1.02	.39	1.03	0.99	.30
High school or GED	0.21	1.01	.84	1.51	0.98	.12
11th grade (reference)						
Medical insurance						
Yes	0.77	0.87	.38	–0.24	0.87	.78
No (reference)						
Perceived stress	0.07	0.08	.36	0.13	0.08	.08
Anxiety control	–0.02	0.02	.24	–0.04	0.02	.07
Depression	0.17	0.06	**.003**	0.17	0.06	**.004**
Substance use	1.90	0.77	**.01**	–0.24	0.68	.72
Status	0.03	0.03	.35	0.04	0.03	.25
Toughness	–0.04	0.05	.42	–0.03	0.04	.46
Anti-femininity	0.13	0.06	**.04**	0.04	0.06	.51

*Note*. β = beta, GED = general equivalency diploma; *SE* = standard error.

Boldface text indicates *p* values <.05.

### Results of Multivariate Poisson Loglinear Regression Analysis Testing the Association Between Dimensions of Masculine Norms at Baseline and Total Number of Mental Health Service Visits in the Prior Year

**Figure 1.**
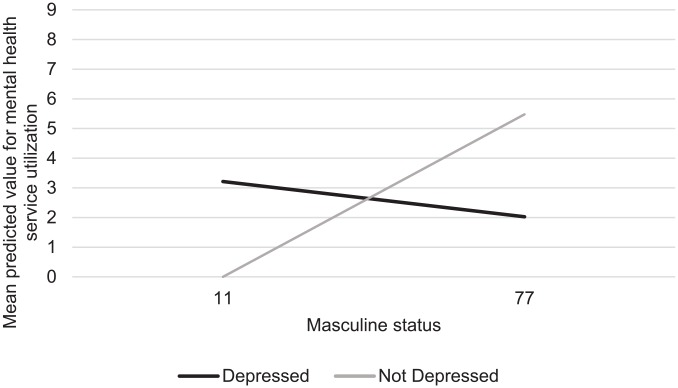
Mean predicted value of mental health service utilization by depression status and endorsement of masculine status norms. *Note*. The graph predicts mental health service utilization by men with the lowest reported masculine status scores versus those with the highest reported masculine status scores (11 and 77), comparing men meeting the criteria for depression and those not meeting the criteria for depression on the Center of Epidemiological Studies-Depression Scale (CES-D) scale.

**Figure 2.**
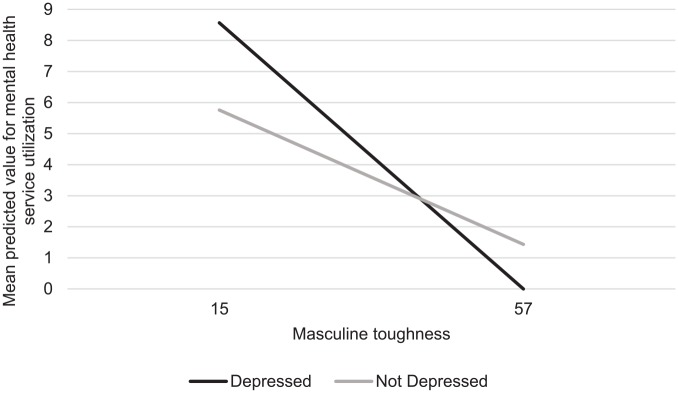
Mean predicted value of mental health service utilization by depression status and endorsement of masculine toughness norms. *Note*. The graph predicts mental health service utilization by men with the lowest reported masculine toughness scores versus those with the highest reported masculine toughness scores (15 and 57), comparing men meeting the criteria for depression and those not meeting the criteria for depression on the Center of Epidemiological Studies-Depression Scale (CES-D) scale.

Controlling for sociodemographic and mental health covariates, statistically significant associations were found between endorsement of masculine status, anti-femininity, and toughness norms and mental health service utilization in the prior year. Detailed statistics are reported in Table 6. Specifically, men who reported greater endorsement of status norms were more likely to see a mental health professional in the prior year (β = 0.07, *SE* = 0.01, *p* < .001), while men who reported greater endorsement of anti-femininity norms (β = −0.03, *SE* = 0.01, *p* = .003) and toughness norms utilized mental health services less (β = −0.06, *SE* = 0.01, *p* < .001). In addition, statistically significant interactions were identified between masculine status and depression status and between masculine toughness and depression status. As depicted in Figure 1, the positive association between status norms and treatment-seeking behavior was stronger for men not meeting the criteria for depression compared to men meeting the criteria for depression (β = −0.07, *SE* = 0.02, *p* < .001). As depicted in Figure 2, endorsing masculine toughness reduced the likelihood of mental health service use for all men; however, this association was stronger for men who met the criteria for depression compared to those who did not (β = 0.04, *SE* = 0.02, *p* = .04). In addition, the following covariates were associated with a greater number of mental health service visits in the prior year: younger age (β = −0.99, *SE* = 0.08, *p* < .001), having medical insurance (β = 2.43, *SE* = 0.46, *p* < .001), meeting the criteria for depression (β = 2.43, *SE* = 1.12, *p* = .01), and less hostility (β = −0.21, *SE* = 0.03, *p* < .001). Men who reported using substances in the prior 30 days were less likely to have reported mental health service utilization in the prior year (β = −1.37, *SE* = 0.19, *p* < .001).

**Table 6.** Results of Multivariate Poisson Regression Analysis Testing the Associations Between Dimensions of Masculine Norms (Baseline) and Total Number of Mental Health Service Visits (Prior Year at Baseline), *N* = 117.

	β	*SE*	*p* value
Age	–0.99	0.08	<.001**
Medical insurance			
Yes	2.43	0.46	<.001**
No (reference)			
Depression			
Yes (CESD score 16+)	2.87	1.12	**.01**
No (CESD score <16)			
Substance use	–1.37	0.19	<.001**
Hostility	–0.09	0.22	<.001**
Status	0.07	0.01	<.001**
Toughness	–0.06	0.01	<.001**
Anti-femininity	–0.03	0.01	.003*
Depression × status	–0.07	0.02	<.001**
Depression × toughness	0.04	0.02	.04*

*Note*. Criterion for depression is defined as a score of 16 or greater on the Center for Epidemiological Studies-Depression (CESD). β = beta, *SE* = standard error.

Boldface text indicates *p* values <.05.

## Discussion

This study sought to understand how the masculine norms of status, toughness, and anti-femininity affect depression symptomology, the related outcomes of substance use and hostility, and mental health service utilization among emerging adult men of diverse backgrounds. This study’s findings add support to a growing body of literature that demonstrates that some masculine norms increase men’s risk of adverse mental health outcomes and reduce the likelihood of psychological support seeking, while others can have a protective effect and are associated with more help-seeking behavior (Iwamoto et al., 2018; Wong et al., 2017). The findings extend the current literature by examining these research questions with a diverse sample of emerging adult men from low-income areas, a group underrepresented in the literature. Emerging adulthood is often characterized by elevated stress culminating in a heightened vulnerability to mental health concerns (Adkins et al., 2009; Ghobadzadeh et al., 2019; Lee & Dik, 2017). This vulnerability is compounded for racial minorities and those from low-income communities, given unique stressors (Diggs & Neppl, 2018; Polanco-Roman et al., 2019; Polanco-Roman & Miranda, 2013), who may be less likely to engage in mental health treatment seeking (Blumberg et al., 2015; Chandra et al., 2009; Cummings, 2014; National Institute of Mental Health, 2019; Parent et al., 2018). In the present sample, masculine status was associated with less depressive symptomology and more treatment seeking, especially for emerging adult men who were not depressed. Masculine toughness was associated with more substance use but less hostility; however, men endorsing masculine toughness were less likely to utilize mental health services, especially when depressed. Finally, anti-femininity norm endorsement was associated with more hostility and less mental health service utilization, but was not related to depression.

Though Wong et al.’s (2017) meta-analysis reported a positive association between the pursuit of status and negative mental health outcomes, in this sample of emerging adult men, those striving for success in work and family life were at lower risk for depression and engaged in more psychological support seeking. Gerdes, Alto, Jadaszewski et al. (2018) report greater courage, self-esteem, self-acceptance, and resilience among men endorsing masculine status, which could be protective against depression. The sample in the current study was uniquely comprised of a racially/ethnically diverse group of emerging adult men, which could also account for differences between this study’s findings on status and Wong et al.’s (2017) review. Though differences by race have not been widely examined, Gordon et al. (2013) reported more preventative behavior and less substance use among young men endorsing masculine status; this effect was more pronounced among young Black fathers compared to their White and Latino counterparts.

By examining the effect of masculine status on mental health service utilization stratified by depression status, this study also supports the idea that the relationship between masculine status and better health outcomes may also be explained through greater engagement in preventative behaviors by racially diverse young men. In this study, emerging adult men endorsing masculine status were more likely to seek mental health services; this relationship was stronger for men who did not meet the depression criteria than for those who did. This finding suggests that men endorsing masculine status norms engage more in primary prevention, that is, seek mental health support before reaching diagnosable depression. Early engagement with mental health services may explain less depression overall among those endorsing masculine status norms.

Men’s socialization to be mentally and physically strong (i.e., toughness) is generally thought to contribute to men’s tendency to hide depressive symptoms and avoid mental health services (Smith et al., 2018). It is possible that socialization to be tough could build resilience and protect against depression. In this sample of emerging adult men, endorsing toughness did have positive mental health effects, including more anxiety control and less hostility, which may be protective against depression. However, toughness was associated with greater substance use at baseline and being less likely to visit a mental health professional. Participants reporting any substance use were also less likely to have utilized health services; it is possible that young men endorsing toughness norms are more likely to self-medicate with substances than seek professional help. Among men who met the criteria for depression, toughness norms had a stronger negative effect on treatment utilization than among those not meeting the criteria for depression. This finding has important implications for our understanding of the intersection of masculinity and mental health, suggesting certain masculine norms may be more detrimental to treatment seeking for men most in need of services. Others researchers report high stigma associated with mental health among emerging adult men, lower income communities, and communities of color (DeFreitas et al., 2018; Gary, 2005; Lindsey et al., 2010; Vogel et al., 2011), which may be exacerbated by endorsement of masculine toughness.

The related masculinity dimension, anti-femininity, was not associated with depression or substance use, but did associate with more hostility and less anxiety control, which carry their own negative implications for psychological well-being and social functioning. Research reports that hostility as well as related constructs captured in our measure of hostility, such as anger, aggression, and irritability, may be male-specific symptoms of depression not captured by typical measures of depression (Call & Shafer, 2018; Martin et al., 2013; Rochlen et al., 2010). Future research on anti-femininity norms would benefit from a comparison of men’s experience/willingness to report male-specific symptoms of depression and those captured in standardized measures of depression (Call & Shafer, 2018; Price et al., 2018). In support of this study’s hypothesis, like toughness, anti-femininity endorsement was associated with less mental health service utilization in the prior year, which may be explained by men’s tendency to feminize depression and counseling (Kilmartin, 2005; Smith et al., 2018).

### Strengths and Limitations

The generalizability of this study is limited to heterosexual young men (18–25 years of age) recruited from low-income neighborhoods in the United States. While the inclusion of racially and ethnically diverse young men is a strength of this study, the study’s sample size limited the ability to make meaningful comparisons across racial and ethnic groups. The importance of intersecting identities (e.g., race, ethnicity, and sexual identity) in the differential display of masculinity and its effect on health is well known (Courtenay, 2000a; Griffith et al., 2013; Hammond, 2012; Williams, 2003). The impact of masculine norms on mental health outcomes observed in the present study may have differed across groups, as reported in other studies (Gordon et al., 2013; Hammond, 2012; Vogel et al., 2011). Future research should seek to further dissect the context- and population-specific effects of masculine norms on depression and psychological help seeking.

This is only the second study after Iwamoto et al. (2018) to use longitudinal data to understand the impact of masculine norms on prospective depressive symptomology. This study strengthens the existing evidence base of primarily cross-sectional studies and extends this literature by examining substance use and hostility as secondary outcomes. However, the examination of the association between masculine norms and health service utilization was cross-sectional, as only 4 participants reported mental health service utilization at 3-month and only 6 participants at 6-month follow-ups, restricting the ability to use longitudinal time points for this outcome. This limitation highlights an area to strengthen with future prospective research designs. Our measure of mental health service utilization was self-reported and therefore subject to recall bias and other forms of response bias such as social desirability.

Another limitation to consider is the possibility that the reported findings are skewed by men’s tendency to underreport typical depressive symptoms and to experience unique symptoms of depression not captured by the CES-D scale (e.g., anger, somatic symptoms, substance use). This may be especially true for anti-femininity; as discussed earlier, these norms were associated with depression’s risk factors in analysis (hostility, less anxiety control), but not with depression. High adherence to traditional masculine norms may increase the likelihood of externalizing depressive symptoms, as reported by Price et al. (2018). U.S. men in their study who endorsed more traditional masculine traits were more likely to endorse externalizing symptoms (anger, substance use) compared to typical internalizing depressive symptoms. Pertinent to the present sample, younger age has been associated with greater externalization of depressive symptoms (Rice et al., 2019). For this reason, hostility and substance use were included as secondary outcomes; however, these measures are not a comprehensive assessment of the full range of possible male-specific symptomology, which also includes emotional suppression, somatic symptoms, and risk-taking (Rice et al., 2013). This highlights a broader methodological weakness of studies on self-reported masculine norms and depression as well as the need for more research to include male-specific measures of depression, such as the Male Depression Risk Scale (MDRS-22; Rice et al., 2013) and Male Depression Scale (Magovcevic & Addis, 2008).

Finally, this study adds new insight into how status, toughness, and anti-femininity norms influence mental health outcomes and service utilization—norms not directly measured in other studies of masculinity and depression. Prior studies include overlapping but different dimensions of masculinity captured by the Conformity to Masculine Norms Inventory (i.e., “Winning,” “Playboy,” “Primacy of Work,” “Risk-Taking,” “Self-Reliance,” and “Emotional Control”) (Mahalik et al., 2003; Parent & Moradi, 2009; Parent et al., 2011). Nevertheless, this examination of masculine norms is far from exhaustive. The MRNS measure used in this study is not without limitations, including measuring only three dimensions and its focus on negative aspects of masculinity, despite this study’s focus on both the negative and positive effects of masculine norms on mental health outcomes. Future research should continue to explore how these and other prominent dimensions of masculine norms affect mental health outcomes and service utilization.

### Implications

Programs to engage men in mental health services can benefit from understanding and incorporating the role of gender and masculinity in programming (Robertson et al., 2018). Strategies put forth include gender-sensitive staff training, the use of gender-sensitive language in public health campaigns and counseling, a “male-positive” approach that recognizes men’s assets and engages men as partners, and activities to improve men’s emotional expression and communication (Robertson et al., 2018). In tandem with other similarly aimed studies (Iwamoto et al., 2018; Wong et al., 2017), this study has implications for the tailoring of outreach messaging and counseling to engage and retain young men in mental health services. This study’s findings reinforce the need to reconfigure masculine toughness norms in order to improve men’s capacity to engage in psychological services, which may increase young men’s substance use, hinder their emotional expression, and exacerbate stigma associated with help seeking (Vogel et al., 2014). The greater negative effect of masculine toughness on help seeking among emerging adult men with the greatest need of depression treatment is an important finding of this study. This finding suggests deconstructing toughness norms may be especially pertinent to engage young men in services who are actually suffering from depression.

Further, including the healthy aspects of masculine status in outreach and counseling may motivate emerging adult men to engage in mental health and preventative services—that is, emphasizing the importance of good mental health for success in men’s goals related to work and family. Sagar-Ouriaghli et al.’s (2019) review identified “content that built on positive male traits (e.g., responsibility and strength)” as one element of male-focused interventions that improves help-seeking behavior. This approach is in line with a broader trend among researchers to understand positive aspects of masculinity (Kiselica et al., 2016; Kiselica & Englar-Carlson, 2010) with the goal of better informing male-centered clinical work (Englar-Carlson & Kiselica, 2013; Mahalik et al., 2012; Spendelow, 2014). Caution must be taken to not simply reinforce existing masculine norms without paying heed to the negative effects these same norms can have on other health behaviors or outcomes (Fleming et al., 2014; Gardner, 2007). As such, mental health researchers and practitioners should explore the adoption of a “gender transformative” approach to mental health, which aims to reconstruct healthier notions of masculinity and move toward gender equity (Dworkin et al., 2015; Fleming et al., 2014). For recommendations beyond tailored program development, Rice et al. (2018) offers a discussion of gaps and areas for growth across policy, theory, and research and evaluation to develop targeted interventions that engage young men in mental health services.

## Conclusions

In this study’s sample of emerging adult men, depressive symptoms were high, with nearly a third of men meeting the criteria for depression at the time of the baseline survey and a quarter of men at 6-month follow-up. Only 14% of the sample had visited a mental health-care provider in the prior year. These findings exemplify the challenge public health practitioners are faced with in engaging young men in mental health services. This study adds support for the role of masculine norms in men’s experience of depression and related outcomes and engagement in mental health services. Masculine status may be protective against depression and promote engagement in preventative mental health services, while masculine anti-femininity and toughness may reduce men’s likelihood of engaging in mental health services, with toughness norms especially detrimental to engagement in psychological health services for men who are suffering from depression. These findings can inform gender-tailored outreach and programming to improve emerging adult men’s engagement with mental health services.
